# Biomechanically and Periodontally-Based Orthodontic Treatment of a Patient with Upper Canine Affected by External Cervical Resorption (ECR): A Case Report

**DOI:** 10.3390/dj11120278

**Published:** 2023-11-30

**Authors:** Marino Musilli, Morena Tina Iuorio, Emanuele Vaia, Enzo Vaia, Luca Ramaglia, Vincenzo D’Antò

**Affiliations:** 1 Private Practice, 84124 Salerno, Italy; marinomusilli@hotmail.com; 2 Department of Neuroscience, Reproductive Science and Oral Science, University of Naples Federico II, 80131 Naples, Italy; mo.iuorio@studenti.unina.it (M.T.I.); emanuele.vaia@unina.it (E.V.); e.vaia@libero.it (E.V.); luca.ramaglia@unina.it (L.R.)

**Keywords:** external cervical resorption, ankylosed upper canine, occlusal plane canting, orthodontic extrusion, case report

## Abstract

(1) Background: Orthodontic treatment may be a potential predisposing factor for ECR. The affected tooth goes to ankylosis, which could lead to a malocclusion. Although teeth severely affected by ECR (class IV Heithersay) are usually extracted, this case report aims to present the use of an ECR class IV upper canine, both as ankylosed to solve the malocclusion and the occlusal plane canting, as well as not ankylosed to correct its ridge defect with orthodontic extrusion. (2) Methods: A 14-year-old male, complaining of an ugly smile and a failed orthodontic attempt to recover an impacted canine, was referred to the orthodontic clinic. He was diagnosed with class II right subdivision, midline deviation, both upper and lower occlusal plane canting, and an upper left canine, previously impacted, showing ECR class IV. The treatment first included canting resolution with a cantilever and a spring, exploiting the anchorage offered by the ankylosed ECR canine. Then, a coronectomy, endodontic treatment, and orthodontic extrusion of that canine were performed to obtain the implant site development. (3) Results: Clinical and radiographic outcomes showed normocclusion and better bony conditions for safer implant placement in the aesthetic zone. (4) Conclusions: The high aesthetics and the periodontal and bony conditions obtained are probably not achievable by other therapeutic alternatives.

## 1. Introduction

Orthodontic treatment aims at aligning teeth or correcting skeletal and functional problems. However, teeth are not always erupted [1], and a patient’s malocclusion can include impacted teeth.

Among the most common reasons for the recovery failure of impacted teeth, together with inadequate anchorage and mistaken directional traction, ankylosis seems to be responsible for 32.4% of failures [2]. However, a retrospective analysis of impacted canine traction failure shows the presence of a typical root resorption pattern [3] that shifts the diagnosis from ankylosis to a condition identified as “Invasive Cervical Root Resorption” (ICRR) or “External Cervical Resorption” (ECR) [3,4].

ECR is an idiopathic and rare external tooth resorption process localized in the area of the cementoenamel junction and connective tissue attachment [4] that gradually involves dentin coronally, apically, and circumpulpally up to the predentine layer but does not involve the pulp until very advanced stages. Unfortunately, scientific literature lacks information regarding this condition; thus, pathogenesis remains unclear. There are several potential predisposing factors that lead to ECR: the aforementioned orthodontic treatment, traumatic injury, internal bleaching, extraction of a neighboring tooth, and chronic periodontitis [5,6,7]. Pathogenetically, it has been linked to any kind of direct trauma on the root cementum that exposes the underlying dentinal tissue to increased odontoclastic action and reduces the activity of bone resorption factors [8,9]. As the dental hard tissue destruction progresses, a cementum-like substance is deposited, leading to a relative increase in tooth resistance that counteracts collapse [8,9]. Therefore, ankylosis followed by replacement resorption and ECR can be confused. Thus, a thorough analysis of traction non-responding teeth to determine a loss of anchorage is essential since ICRR loss of tooth mobility is only partial, related to the localized resorption [3].

Depending on the extension and localization of resorption, the severity of ECR can be divided into four different classes, according to Heithersay [10]. While different treatment modalities have been proposed for the initial stages, teeth with advanced ECR lesions are candidates for extraction [3].

Although orthodontic treatment could have different endpoints, symmetry is considered a key aspect in achieving an ideal smile [11]. Unfortunately, treating asymmetries often generates different asymmetries in other planes of the space [12]. The same scenario could take place in cases of undiagnosed ankylosis, resulting in unexpected teeth movements.

Among asymmetries, occlusal canting could be listed as one of the most challenging clinical situations for orthodontists. Different segmented mechanics have been proposed in order to give a new symmetry to the occlusal plane, but they generate a step between anterior and posterior teeth as a side effect. In order to reduce the weight of side effects, a novel approach combining a simple auxiliary canting spring with a continuous archwire to correct canted incisal planes was proposed by M. Musilli [13].

The aim of the present case report is to describe a valid orthodontic, biomechanically and periodontally-based treatment for a severe ECR case of an impacted canine associated with a severely canted occlusal plane.

## 2. Patient Information—Case Presentation

A systemically healthy 14-year-old male patient presented at the orthodontic clinic. His chief complaints were an ugly smile and a failed orthodontic attempt to recover an impacted upper left canine. The patient reported that he had previously undergone surgical exposure of the upper left canine to move it to its correct occlusal position by means of an orthodontic appliance. The patient’s case report is presented following the CARE guidelines checklist [14] in Appendix A.

### 2.1. Clinical Findings

**Figure 1:**
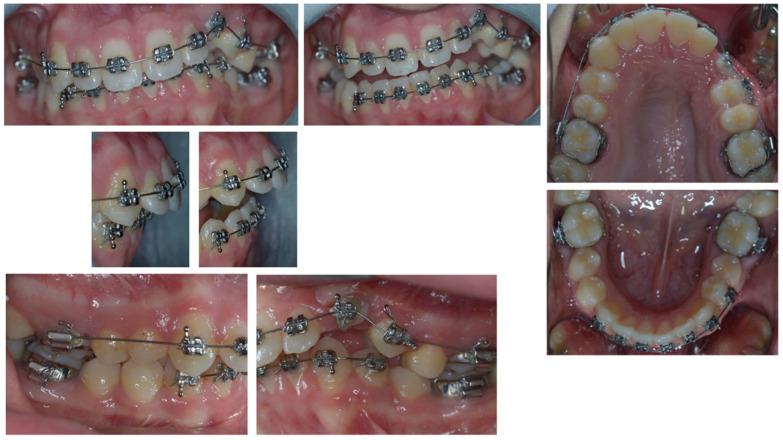
Intra-oral patient baseline presentation (with multibrackets fixed appliance elsewhere used to recover the impacted canine).

**Figure 2:**
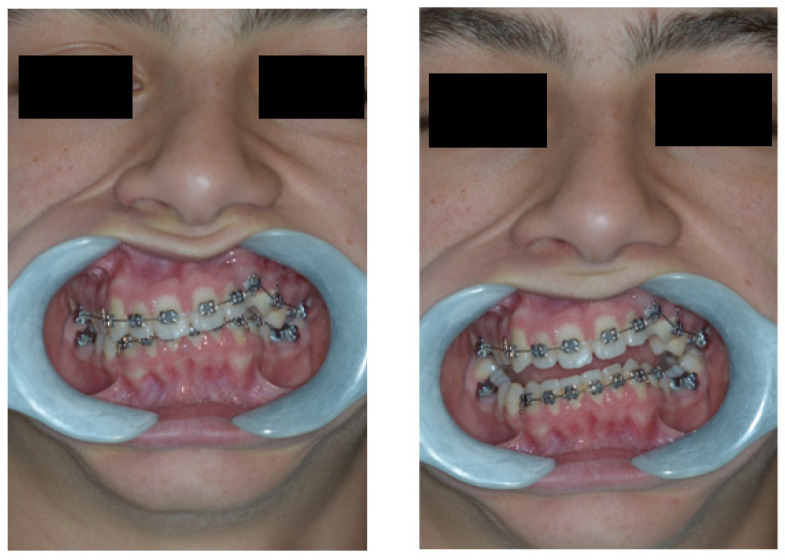
Extra-oral patient baseline presentation (with multibracket fixed appliance elsewhere used to recover the impacted canine).

On examination, the patient was periodontally healthy and did not show any carious lesions. He presented class II right subdivision type 1 with lower midline deviation [15], both lower and upper occlusal plane canting, and an upper left canine that was partially erupted but ankylosed. He had an intraoral multibracket fixed appliance that, referring to the anamnesis, was elsewhere used to recover the impacted canine (Figure 1 and Figure 2). That multibracket appliance with slot “0.018 × 0.025” was probably the Roth technique.

### 2.2. Radiographic Findings

**Figure 3:**
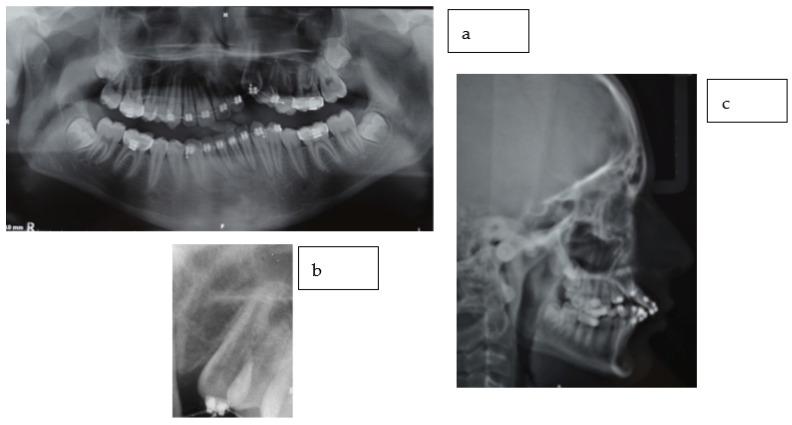
X-rays: orthopantomography (**a**), intraoral X-ray (**b**), teleradiography (**c**). (**a**,**b**) showed a radio transparency on the distal surface of the upper left canine. Cantings were also evident in (**a**,**c**). No other anomalies could be detected.

An orthopantomographic X-ray showed a radio-transparency on the distal surface of the upper left canine. Cantings were also evident. No other anomalies could be detected (Figure 3).

### 2.3. Diagnostic Assessment

Radiographic data, together with the anamnesis and the clinical appearance, were compatible with the diagnosis of ECR of the upper left canine. The diagnostic assessment revealed that, most likely, the orthodontic force and surgical disimpaction activated the canine’s ECR grade 4 Heithersay [10] and its consequent ankylosis.

The delay in achieving a correct diagnosis was responsible for the canting due to the perpetuation of the ankylosed canine, determining an intrusion of adjacent teeth rather than the canine’s extrusion. Lower canting was probably due to the use of vertical elastics from the upper left canine to the lower arch teeth or to indiscriminate alignment [16] without occlusal vertical control of the lower left side (as a secondary consequence of upper antagonist teeth intrusion).

## 3. Therapeutic Interventions

The main aim of the treatment was to solve the severe malocclusion. The class 4 ECR was attributed to a hopeless prognosis for the upper left canine. However, an eventual extraction could not be immediately followed by an implant placement since the patient was still growing.

Thus, the ECR tooth was planned to be used in a twofold way. First, the ankylosis was used to solve the upper canting by using the element for maximum anchorage. For this purpose, it was connected to a cantilever activated in intrusion for the first quadrant directly from the canine. To solve lower arch canting, a modified Ricketts utility arch similar to a canting spring was used [13], working without molar tip activations but only with vertical activation on the front teeth.

**Figure 4:**
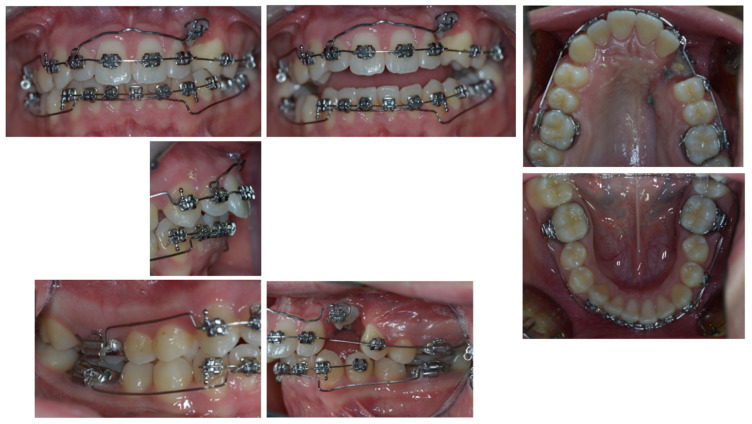
The end of the canting resolution phase is shown intra-orally. Here, the ECR upper left canine was located apical to its ideal position and far from the occlusal plane.

**Figure 5:**
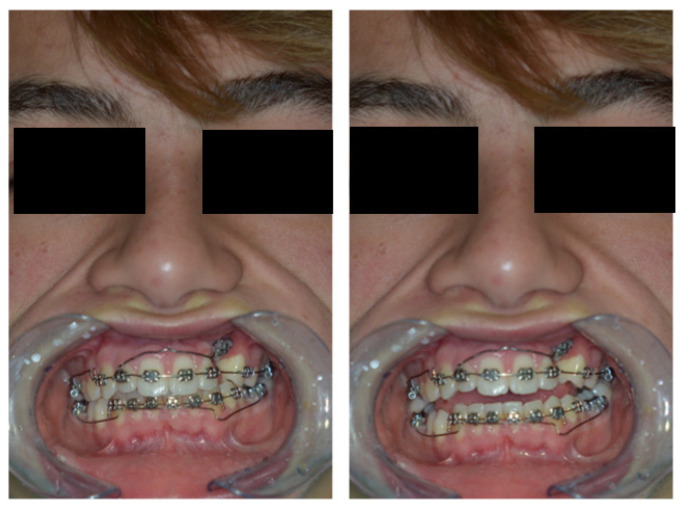
The end of the canting resolution phase extra-orally is shown. The ECR upper left canine was located apical to its ideal position and far from the occlusal plane.

At the end of the canting resolution phase (Figure 4 and Figure 5), the ECR upper left canine was located apical to its ideal position and far from the occlusal plane, creating a vertical discrepancy between the actual and ideal bone levels that would have worsened with its extraction. With the final aim of placing an implant and avoiding the need for invasive horizontal and vertical ridge reconstructions, the tooth underwent an orthodontic extrusion when the anchorage outlived its function.

Several scientific articles and case reports describe the role of orthodontic extrusive remodeling in the enhancement of soft and hard tissue profiles prior to implant placement [17,18,19,20,21,22,23,24,25,26,27,28,29,30,31,32,33,34,35,36].

**Figure 6:**
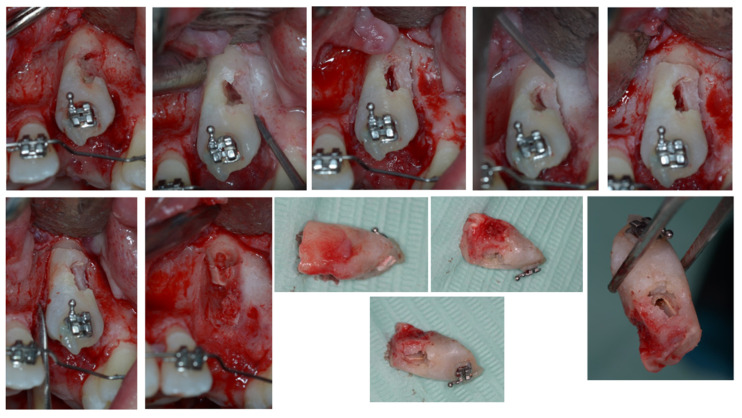
Surgical canine exposure to remove the damaged ankylosed distal surface with coronectomy.

**Figure 7:**
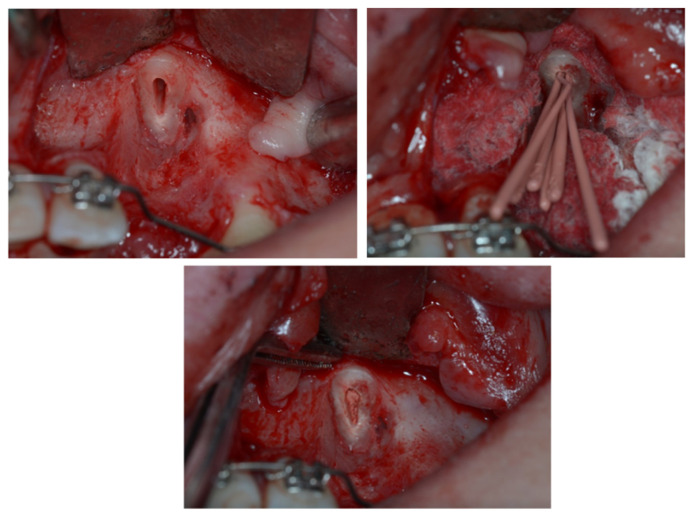
Endodontic treatment of 2.3 element residual root.

**Figure 8:**
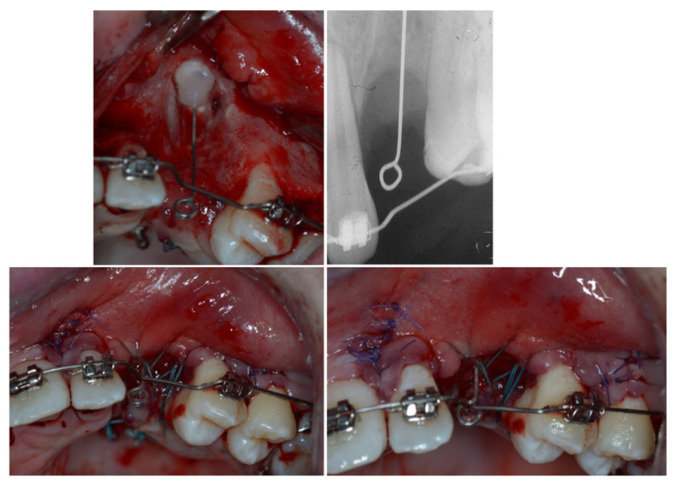
Cantilever bonded to the residual root.

In this case, the upper left canine was surgically exposed by means of a trapezoidal full-thickness flap. Then, it underwent a coronectomy extending apically to the ECR lesion in order to remove the damaged ankylosed distal surface (Figure 6) and was subsequently treated endodontically (Figure 7). Before the flap suture, a cantilever was bonded to the residual root (Figure 8). 

**Figure 9:**
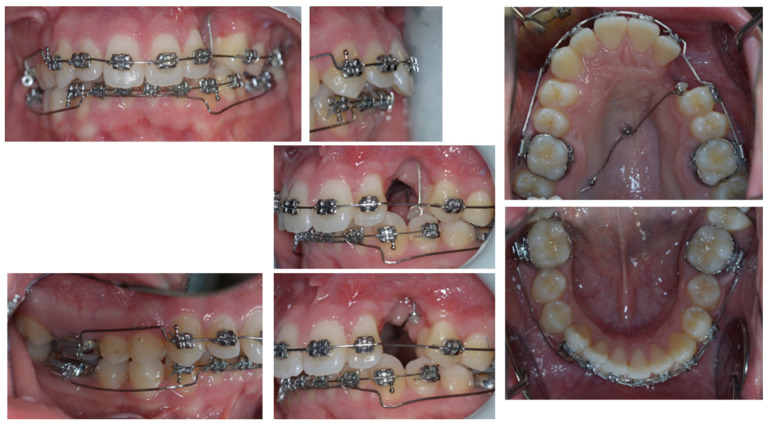
The extrusion system with the Elgiloy cantilever was inserted in two palatal mini-screws and connected to the canine residual root.

**Figure 10:**
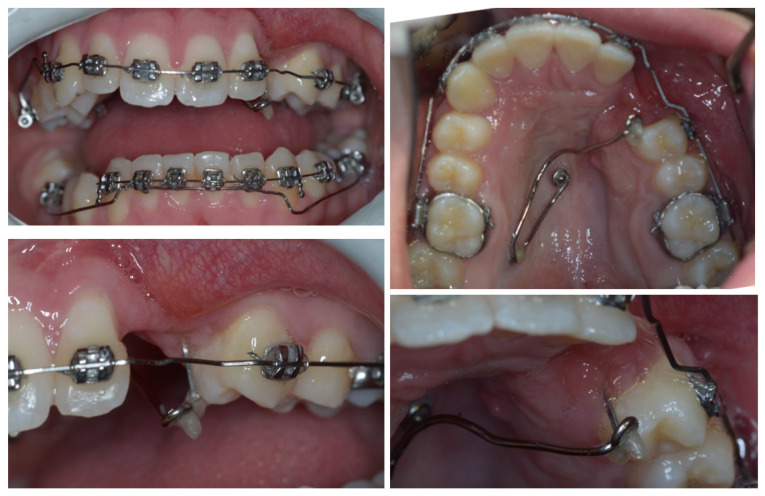
A 0.9 mm cantilever in stainless steel replaced the first one to increase the extrusion at the orthopaedic force of 500 g.

**Figure 11:**
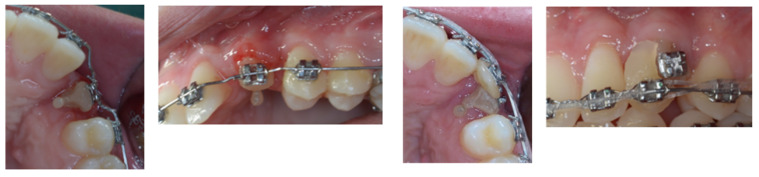
As soon as the residual root erupted, the canine was orthodontically splinted with a small composite veneer for aesthetic reasons.

The extrusion was first planned using a 0.016 × 0.022-inch Elgiloy cantilever inserted in two palatal -, activated in extrusion, and connected to the canine residual root (Figure 9). However, no movement happened with the normal orthodontic extrusive force delivered by this cantilever, likely due to a paracellar block consequent to incomplete removal of the ankylosed area. Thus, a cantilever 0.9 mm in stainless steel replaced the first one to increase the extrusion at the orthopedic force of 500 g (Figure 10). As soon as the residual root erupted, it was orthodontically splinted with a small composite veneer for aesthetic reasons (Figure 11).

**Figure 12:**
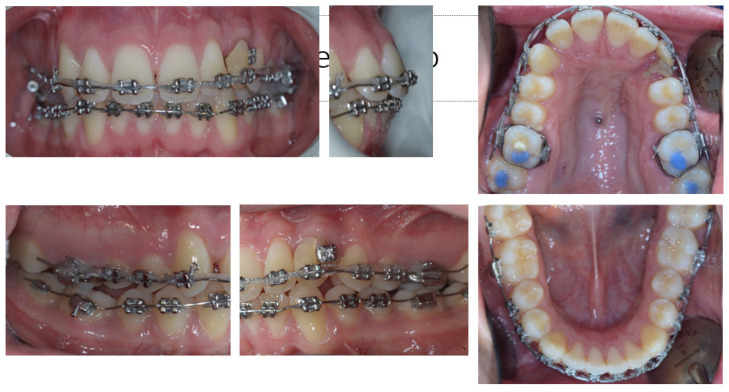
Malocclusion of class II left subdivision to correct with class II elastics.

**Figure 13:**
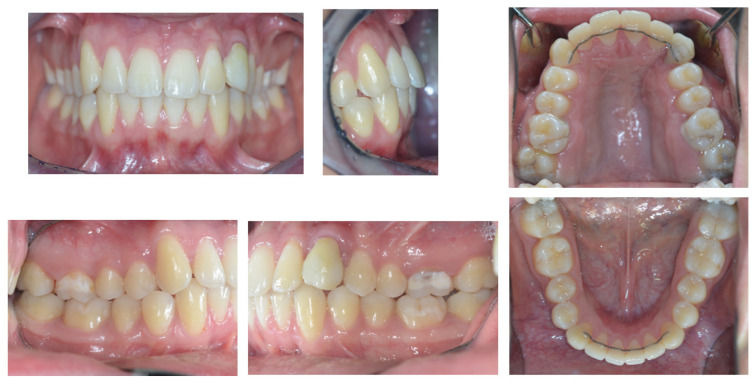
Final normo-occlusion.

**Figure 14:**
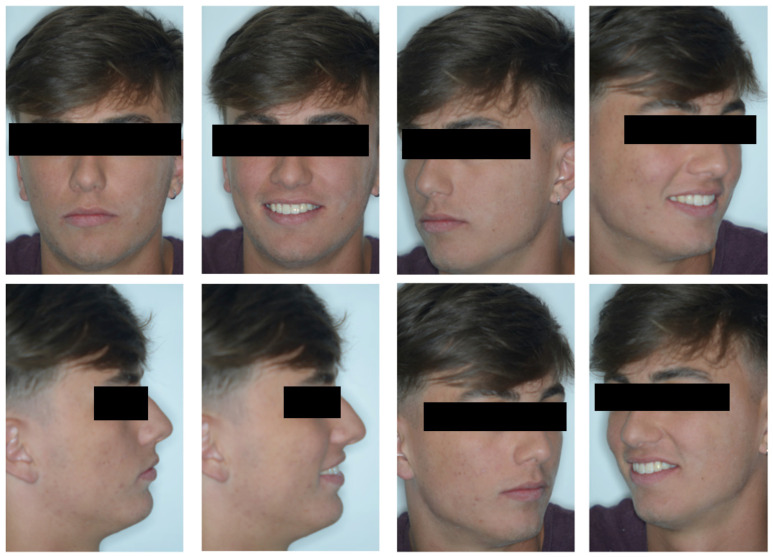
Good aesthetic facial and profile.

**Figure 15:**
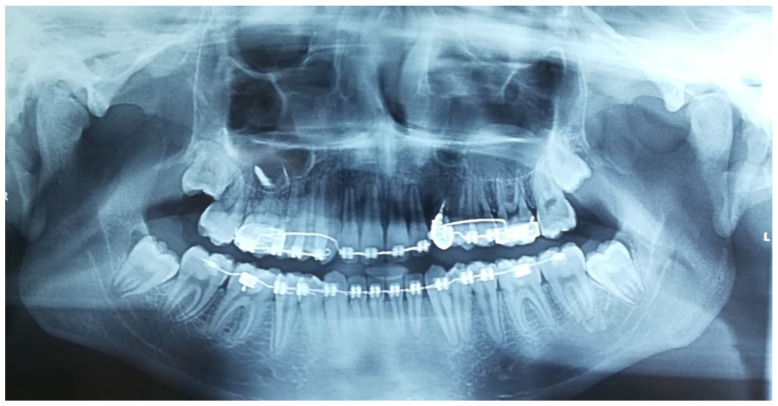
Normal panoramic X-ray features.

The last step of the orthodontic treatment aimed at the resolution of the second class subdivision (Figure 12) by using a multibracket fixed appliance with slot “0.018 × 0.022” in Ricketts technique, by means of class II elastics, leading to the final normal-occlusion (Figure 13), good aesthetic facial and profile (Figure 14), and normal panoramic X-ray features (Figure 15).

## 4. Follow-Up and Outcomes

The upper cantilever activated in intrusion directly from the canine solved the upper canting. A lower modified Ricketts utility arch, similar to a canting spring, solved the lower canting. The upper cantilever and the lower modified Rickets utility completely corrected the upper and lower canting, respectively, in four months. Then, the forced orthodontic extrusion was performed with an SS cantilever, leaving 500 g of orthopedic force, reaching the residual root eruption in five days. Post-surgical healing was uneventful, allowing the clinician to remove the sutures at 14 days. A further control visit a month thereafter confirmed the absence of any complications. The orthodontic splint of the canine’s residual root allowed clinicians to control its mobility and not only maintain but also increase the alveolar bone height at that site. However, the site development due to the residual root extrusion was not only quantitative (volume increase) but also qualitative. With the periodontal attachment being moved apically by the surgery and its coronal movement, it was possible to gain keratinized tissue. As shown by the comparison of pre-surgical and final pictures, the vestibular gingival tissue changed from an absence to a height comparable with the adjacent teeth. 

At the end of the orthodontic fixed treatment with the multibracket fixed appliance and with class II elastics, the patient reached normo-occlusion. He showed midline coincidence, normal overjet, normal overbite, and class I molar and canine relationship. 

At the end of orthodontic treatment, upper and lower fixed retainers were placed. The extruded left canine residual root underwent conservative prosthetic treatment in order to replace the crown and maintain the bony peaks and aesthetics. The patient undergoes semestral follow-ups and oral hygiene sessions while waiting to reach a proper age for implant placement in the upper left canine site.

## 5. Discussion

The present case report illustrates the treatment of a young patient presenting II class II right subdivision with both lower and upper severe occlusal plane canting and midline deviations associated with the upper left canine partially erupted and suffering from a class IV Heitersay ECR. 

In this case report, the orthodontic therapy was carried out with the following objectives: solve the problems that arose from the previous treatment; improve and regulate the shape of the arches; create a correct occlusion; and, finally, prepare the patient for a future, safer, less invasive, and more predictable implant-prosthetic rehabilitation. It was necessary to remove the canine crown with the only aim not to avoid the implant but to carry out a treatment that could develop the implant site, guaranteeing better quality and quantity of the alveolar bone. 

Only the part of the canine with root resorption and ankylosis (affected by ECR) was removed, leaving and using the most apical healthy root portion to carry out the orthodontic extrusion. Alternative treatment for the ankylotic tooth could have included the following: (a) luxation or distraction [37,38], but in the case presented, the ankylosis area was too extensive, leading to a high degree of difficulty or impossibility of the intervention; (b) osteotomy cuts [37], but in that case, the position of the ECR canine was too high; (c) extraction of the tooth, with a consequent bone deficit and subsequent worsening, due to the age of the patient and to the extrusive movements to be carried out on the teeth adjacent to the ankylotic one. The extraction would have been followed at the end of growth by a bone graft operation (probably with sampling from other areas in order to manage a significant extension of the defect found at the end of growth) and subsequently by difficult implant prosthetic replacement or prosthetics on dental elements.

However, the biological and histological foundations that led the authors to develop the approach performed appear in periodontal research carried out on monkeys [39].

Class IV ECR teeth are considered hopeless and are thus extracted and eventually replaced [40]. The present case report points out specific characteristics of ECR that could make affected teeth precious in some treatment plans.

According to histological findings, both in vital and endodontically treated teeth, ECR pathologically undergoes three phases, ending with the deposition of a bony-like tissue inside the newly-formed cavities [7,41].

The observed clinical ankylosis is, histologically, only localized at the lesion site, and it deeply differs from what is normally seen in ankylotic teeth, where it extends all over the root. Therefore, teeth affected by advanced stages of ECR could be used for maximum orthodontic anchorage, as well as a normal tooth to be moved throughout the affected site if bypassed by coronectomies.

In this specific clinical case, the residual root extrusion obtained after the surgery allowed the site to develop both quantitatively and qualitatively. The extrusion created an edentulous ridge showing optimal bony conditions, certainly better than what would have existed if the canine had been extracted in that young patient. In fact, both the canine extraction and residual alveolar growth in that patient would have caused a significant bone deficit in that site, compromising the possibility and success of further implant-prosthetic rehabilitation. Thus, the extrusion being responsible for the site bone development will enable the clinician to avoid the need for more complex, invasive, and less predictable reconstructive surgeries to allow a correct implant placement and reach a desirable aesthetic result.

On the other hand, the development of an adequate keratinized tissue height, thanks to the coronal movement of the new periodontal attachment, could camouflage the presence of the future implant aesthetically without the need for soft tissue grafting. Furthermore, if maintained during the implant-prosthetic procedures, the presence of the keratinized tissue is currently considered a positive prognostic factor for implant health by most experts.

The complexity of the orthodontic treatment could have faced the severe upper and lower occlusal plane canting by adopting a few orthodontic alternatives for this case: (1) the use of continuous, preactivated arch-wires according to the indications of Eric Liou [42]; the use of levers with rear anchorage, described by Nanda [43,44]; the use of an activated utility arch with tip back of opposite direction on a three-part segmented arch, suggested by Dr. Roberto Ciarlantini; or the use of skeletal anchorage in the alveolar vestibular region of the over-erupted areas of the upper and lower arches.

The pre-activation for canting correction described by E. Liou is, basically, made up of opposite curvatures activated in the arch-wire posterior sectors (accentuated-Spee curve on one side and anti-Spee curve on the other), hoping that they can create an intrusive effect in a canine region of one side and an extrusive effect in the opposite canine region of the same arch. This method was discarded, both because it relied too much on posterior dental anchorage, already particularly stressed by the previous therapeutic phases, and because it did not sufficiently guarantee that the movement would take place mainly in the anterior sector, based on the concept of indiscriminate alignment. The solution proposed by Nanda and Uribe consists essentially of segmenting the lower arch into two or three parts, combined with the use of one or two cantilevers, in order to activate vertical forces (intrusive/extrusive) on the anterior region (between the canines). This method was not chosen because, although it is very useful in other cases of canting, in this case, it would have created too important vertical steps between the posterior segments and the anterior segment. For the same reason, the mechanics proposed by Roberto Ciarlantini were not considered useful.

The use of inter radicular skeletal anchorage, as indicated in the third alternative option, would have been excellent for the patient, but it was discarded to avoid the use of excessive and unnecessary mini-screws; strong skeletal anchorage already existed in the upper arch, consisting of ankylosed 2.3 element. In any case, we would have used the mini-screws later for the forced eruption of the 2.3 element root. For the lower arch, a system similar to the canting spring was chosen.

This choice allowed the clinician to start the therapy immediately without providing for changes in the technique, the change of the brackets, or the reintegration of detached brackets. The upper arch cantilever gave only intrusive force on the most extruded point of the arch, while the lower utility arch, preactivated as a canting spring and overlayed onto the pre-existing continuous arch, developed four vertical forces of about 30 g each. Two of them, opposite in direction, were in the anterior sector, at the level of the anterior steps of the utility arch, creating a moment suitable for the correction of the occlusal plane of the frontal teeth. The other two forces were posterior vertical forces of similar intensity, creating a moment of opposite direction to that one realized in the anterior sector. The individual posterior forces released at the height of the molars were considered to be of such intensity as to be well controlled by the occlusion.

## 6. Conclusions

Thanks to their histologic features, teeth affected by ECR can be considered partly ankylosed. Clinically, this means that they could be orthodontically regarded as ankylotic or not, depending respectively on the maintenance of the whole tooth or exclusively the part apical to the lesion. Therefore, they can be used for maximum orthodontic anchorage, as well as extruded to preserve or increase bony profiles in the same clinical case.

On these bases, occlusal canting was solved, thanks to ankylosed canine with ECR and then an orthodontic extrusion, following the removal of the 2.3 element ankylotic area, was performed to prepare the site for future implant placement. The high aesthetics and periodontal and peri-implant health obtained would probably not have been achievable by other therapeutic alternatives.

## 7. Patient Perspective

The patient is now awaiting the right age to insert the implant. Even if the forced extrusion may not have increased the thickness of the alveolar bone because the root portion used for the implant site development had a reduced size and, above all, a reduced section, currently, there is good height and regularity of the dental arches, both in aesthetics and function. These conditions will greatly facilitate the subsequent implant-prosthetic phases.

## 8. Informed Consent

Finally, regarding CARE guidelines, we obtain the signed informed consent of the patient to publish online his case in an open access format paper. We believe that authors have an ethical duty to obtain informed consent from the patient to publish patient information in a case report. Consent becomes informed when the patient or a relative reads the case report and approves its contents, as happened in this case.

## Figures and Tables

**Figure A1:**
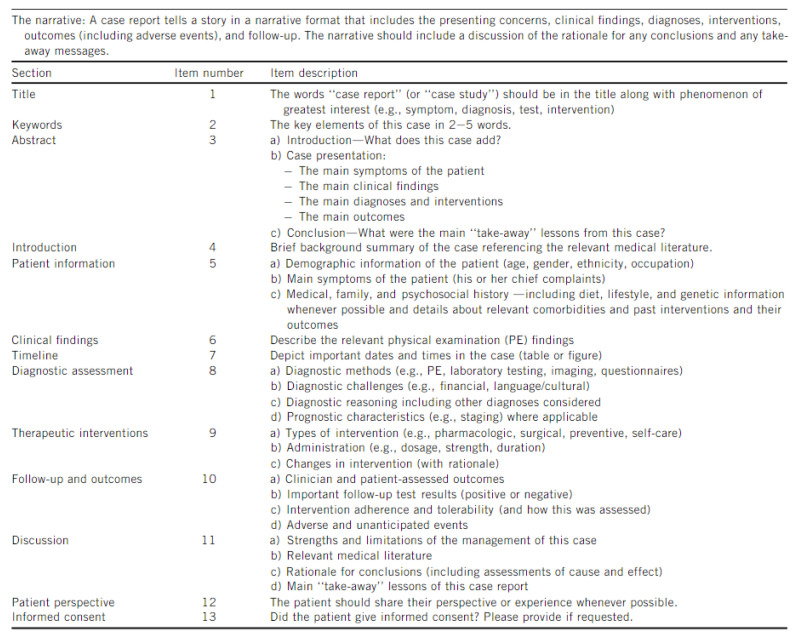
CARE Guidelines Completed Checklist.

## Data Availability

The data presented in this study are available on request from the corresponding author.
